# A New Method to Investigate How Mechanical Loading of Osteocytes Controls Osteoblasts

**DOI:** 10.3389/fendo.2014.00208

**Published:** 2014-12-09

**Authors:** Marisol Vazquez, Bronwen A. J. Evans, Daniela Riccardi, Sam L. Evans, Jim R. Ralphs, Christopher Mark Dillingham, Deborah J. Mason

**Affiliations:** ^1^Arthritis Research UK Biomechanics and Bioengineering Centre, School of Biosciences, Cardiff University, Cardiff, UK; ^2^Institute of Molecular and Experimental Medicine, School of Medicine, Cardiff University, Cardiff, UK; ^3^Division of Pathophysiology and Repair, School of Biosciences, Cardiff University, Cardiff, UK; ^4^Institute of Mechanical and Manufacturing Engineering, School of Engineering, Cardiff University, Cardiff, UK; ^5^School of Psychology, Cardiff University, Cardiff, UK

**Keywords:** osteocyte, osteoblast, 3 dimensional, co-culture, model, loading

## Abstract

Mechanical loading, a potent stimulator of bone formation, is governed by osteocyte regulation of osteoblasts. We developed a three-dimensional (3D) *in vitro* co-culture system to investigate the effect of loading on osteocyte–osteoblast interactions. MLO-Y4 cells were embedded in type I collagen gels and MC3T3-E1(14) or MG63 cells layered on top. Ethidium homodimer staining of 3D co-cultures showed 100% osteoblasts and 86% osteocytes were viable after 7 days. Microscopy revealed osteoblasts and osteocytes maintain their respective ovoid/pyriform and dendritic morphologies in 3D co-cultures. Reverse-transcriptase quantitative polymerase chain reaction (RT-qPCR) of messenger ribonucleic acid (mRNA) extracted separately from osteoblasts and osteocytes, showed that podoplanin (E11), osteocalcin, and runt-related transcription factor 2 mRNAs were expressed in both cell types. Type I collagen (*Col1a1*) mRNA expression was higher in osteoblasts (*P* < 0.001), whereas, alkaline phosphatase mRNA was higher in osteocytes (*P* = 0.001). Immunohistochemistry revealed osteoblasts and osteocytes express E11, type I pro-collagen, and connexin 43 proteins. In preliminary experiments to assess osteogenic responses, co-cultures were treated with human recombinant bone morphogenetic protein 2 (BMP-2) or mechanical loading using a custom built loading device. BMP-2 treatment significantly increased osteoblast *Col1a1* mRNA synthesis (*P* = 0.031) in MLO-Y4/MG63 co-cultures after 5 days treatment. A 16-well silicone plate, loaded (5 min, 10 Hz, 2.5 N) to induce 4000–4500 με cyclic compression within gels increased prostaglandin E_2_ (PGE_2_) release 0.5 h post-load in MLO-Y4 cells pre-cultured in 3D collagen gels for 48, 72 h, or 7 days. Mechanical loading of 3D co-cultures increased type I pro-collagen release 1 and 5 days later. These methods reveal a new osteocyte–osteoblast co-culture model that may be useful for investigating mechanically induced osteocyte control of osteoblast bone formation.

## Introduction

Osteocytes are by far the most abundant bone cell type (90–95% of all bone cells (1), forming a network of cells, connected by long cell processes that extend along canaliculi within the mineralized bone matrix. The adult human skeleton is continually being remodeled by bone forming osteoblasts and bone resorbing osteoclasts, whose activities are balanced in healthy individuals. Osteocytes are thought to integrate hormonal, growth factor, and mechanical stimuli to influence control of bone remodeling.

*In vivo*, osteocytes increase transcriptional and metabolic activities in response to short loading periods (2, 3), increase their dentin matrix protein 1 (DMP1) and matrix extracellular phosphoglycoprotein (MEPE) expression, controlling bone matrix mineral quality (4, 5), increase insulin growth factor 1 (IGF-1), and related proteins involved in mechanically induced bone formation (6, 7), and stimulate nitric oxide (NO) production (8), an early mediator of mechanically induced bone formation (9). Osteocyte abundance, morphology, position within bone, and ability to form an extensive network are ideally suited to this mechanoresponsive role (10–14). Osteocytic processes and primary cilia detect mechanical stimuli (15–19) whereas, proteins involved in the connection of osteocytes to surrounding cells and/or the extracellular matrix (ECM), like focal adhesions, connexin 43 (CX43), and integrins, are involved in osteocyte response to mechanical stimuli (20, 21). Mechanically loaded osteocytes regulate osteoblast activity through various mechanisms including the downregulation of sclerostin (SOST) expression (22, 23), release of NO, which has an anabolic effect on osteoblast activity (9), and release of prostaglandin E_2_ (PGE_2_), which regulates osteoblast proliferation and differentiation (24). Whilst the methodology developed here focuses on osteocyte control of osteoblasts, it is clear that osteocytes regulate other cells activities in response to load (25–27).

A major problem with investigating osteocytes is the difficulty in isolation and culture of these cells *in vitro*. Studies using sequential digestion of bone to enrich for osteocytes have proved difficult and, so far, limited to chick (28–30), rat (31), and mouse (32). Mouse cell lines representing late osteoblast/early osteocytes (MLO-A5) (33) and osteocyte-like (MLO-Y4) (33, 34) cells have been developed to facilitate *in vitro* investigation of osteocytes, but these are routinely cultured in monolayer on type I collagen-coated plastic. More recently the IDG-SW3 mouse derived cells have been shown to replicate osteoblast-to-late osteocyte differentiation under both two-dimensional (2D) and three-dimensional (3D) collagen culture conditions *in vitro* (35).

There have been very few publications on co-culture of osteoblasts and osteocytes, despite the known physiological interactions between these cell types. Taylor et al. (36) describe a co-culture system in which the two cell types are grown in 2D, either side of a semi permeable cell culture insert membrane. Stimulation of the osteocyte layer by fluid shear enhanced alkaline phosphatase (ALP) expression by the osteoblasts, an effect at least partially dependent on cell–cell contact and gap junction communication (36). This system is useful but does not allow osteocytes to form a 3D network. The three-dimensionality of osteocyte environment is important; firstly embedding primary osteoblasts within 3D matrices induces differentiation to osteocyte-like cells *in vitro* (37), recapitulating the *in vivo* differentiation pathway, and secondly it facilitates a more realistic model of a 3D lacunocanalicular system (LCS) of cells that can be subjected to appropriate mechanical cues.

*In vitro*, 3D bone models where bone cells are embedded in type I collagen gels have not been used to investigate osteocyte loading or osteocyte–osteoblast interactions (38–42). 3D cultures made out of polybicarbonate membranes (37) and scaffolds (43–46) do not embed cells within a 3D matrix, but instead attach them to the scaffold surface and therefore do not accurately capture the environment of an osteocyte within bone. Whilst these systems have proven the feasibility of reproducing the synthesis of an organized matrix (44) and cell-mediated matrix degradation (47–49), there are no models that co-culture osteoblasts and osteocytes in 3D under mechanical stimulation. This highlights a major gap in the understanding of the interactions that lead to mechanically induced bone formation.

**Figure 1:**
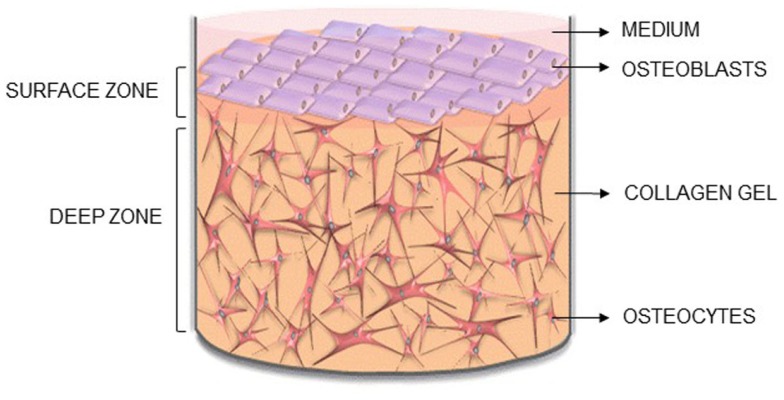
**Novel 3D osteocyte–osteoblast co-culture model**. Diagram of the 3D *in vitro* model indicating the surface and deep zone, and positions of the surface osteoblasts and embedded osteocytes.

Here, we describe the methodology for a new 3D co-culture model, cultured within a custom built multi-well silicone loading plate, to investigate how mechanical loading of osteocytes regulates osteoblast function. MLO-Y4 cells were cultured within type I collagen gels, with an osteoblast-like cell line [MC3T3-E1(14) or MG63] layered on top of the gel (Figure 1). Both osteoblasts and osteocytes maintain cell viability, morphology, and phenotype when cultured in 3D co-cultures and express CX43, a component of network formation. These co-cultures resulted in anabolic responses when stimulated with bone morphogenetic protein 2 (BMP-2) or mechanically loaded. This model will be useful in elucidating osteocyte-driven mechanical mechanisms that regulate bone formation.

## Materials and Methods

### Cells

MLO-Y4 cells were a kind gift from Professor Lynda Bonewald, University of Missouri-Kansas City, USA. MC3T3-E1(14) and MG63 cells were obtained from the European Collection of Cell Cultures, Salisbury, UK.

MLO-Y4 cells (34) were cultured on collagen-coated flasks (rat tail tendon type I collagen, 0.15 mg/mL in 0.02 N glacial acetic acid) in alpha minimum essential medium (αMEM, Invitrogen) supplemented with 2.5% Heat Inactivated Fetal Bovine Serum (HIFBS, Invitrogen) and 2.5% Heat Inactivated Newborn Calf Serum (HINCS, Invitrogen) (50). MC3T3-E1(14) cells were cultured in αMEM supplemented with 10% FBS (Invitrogen) (51). MG63 cells were cultured in Dulbecco’s Minimum Essential Medium (DMEM, Invitrogen) and supplemented with 5% FBS (Invitrogen). All three cell lines were supplemented with 100 U/mL penicillin and 100 μg/mL streptomycin and grown at 37°C in 5% CO_2_. At 70–80% (MLO-Y4) or 80–90% [MC3T3-E1(14) and MG63] confluency, cells were sub-cultured by treating with trypsin/ethylenediaminetetraacetic acid (EDTA) (0.25% w/v of each; Invitrogen).

### 3D co-cultures

MLO-Y4 cells were incorporated within type I collagen gels and either MC3T3-E1(14) or MG63 cells layered on top. Rat tail tendon type I collagen (Sigma, in 7 mM glacial acetic acid) was mixed 4:1 with 5X MEM (Invitrogen) containing 11 g/L sodium bicarbonate on ice and neutralized [1 M tris(hydroxymethyl)aminomethane (Tris) base, pH 11.5] to give 2–2.6 mg/mL type I collagen gels. MLO-Y4 cells (1.5 × 10^6^ cells/mL gel) diluted in αMEM (<10% of total gel volume) were added to the collagen on ice and 500 or 250 μL distributed into 24 or 48-well plastic plates, respectively for polymerization at 37°C for 1 h. MC3T3-E1(14) or MG63 cells (1.5 × 10^5^ cells/well) in DMEM with 5% FBS (MG63) or 5% dialyzed FBS (DFBS) [MC3T3-E1(14)] were applied onto the surface of each gel after 1 h and incubated at 37°C for up to 1 week (Figure 1). Medium was changed after 24 h and every 2 days thereafter. To test cell responses, co-cultures were treated with human recombinant BMP-2 (250 ng/mL, Peprotech) for 5 days.

### Cell viability

Co-cultures grown in plastic plates were rinsed with phosphate buffered saline, pH 7.3 (PBS), incubated with 1 μM ethidium homodimer (Invitrogen) in serum free medium for 2 h at 4°C and then for a further 2.5 h at 37°C before washing overnight at 37°C in normal culture medium with gentle agitation. Positive controls co-cultures were freeze-thawed at −20°C three times, before treatment. For cell death analysis of the surface zone, confocal microscopy was performed directly on whole co-cultures. Samples were scanned using appropriate excitation and emission settings for simultaneous recording of 4′,6-diamidino-2-phenylindole (DAPI) [358 nm Excitation (Ex_(max)_); 461 nm Emission (Em_(max)_)] and ethidium homodimer [590 nm Ex_(max)_; 617 nm Em_(max)_]. Samples were optically sectioned, over five defined arbitrary regions per gel quarter, using a x10 objective lens with 2.32 zoom. 5 μm step size z-stack optical sections were reconstructed using Leica Confocal Software. Maximum intensity models were prepared showing detail of the surface zone. Counts were made of DAPI (blue) labeled nuclei (to give total number of cells) and ethidium homodimer and DAPI (purple) co-labeled nuclei (to give number of dead cells).

For deep zone viability, cultures were fixed with 1% paraformaldehyde (Sigma) in 0.05 M PBS for 30 min at 4°C and then washed in PBS. Some were labeled whole for filamentous actin and type I pro-collagen (see below). Cultures were infiltrated with 50% OCT compound (Tissue Tek) in PBS overnight at 4°C and then frozen in fresh OCT compound onto cryostat stubs using dry ice. Cryosections were cut at 20 μm using a Bright OTF5000 cryostat and collected on Polysine slides (VWR). Five random slides of each co-culture containing 4–6 sections each were mounted in Vectashield mounting medium with DAPI as a nuclear counterstain. One random section from each slide was observed under epi-fluorescence as above, and 10 random fields of view using the x20 objective photographed for each section under both DAPI and ethidium homodimer illumination. Counts were made as above.

### Microscopy and imaging of cells and cell markers

Osteocyte and osteoblast morphology was assessed in live cultures grown in plastic plates with a Leica DMRB inverted microscope equipped with a Moticam 2000 digital camera. For labeling procedures, 3D cultures were treated, fixed, frozen, and cryosectioned as previously outlined.

Filamentous actin was labeled by incubating fixed intact cultures or sections with 5 μM Atto488 conjugated phalloidin (Sigma) in PBS containing 0.1% tween 20 (PBST; Sigma) for 40 min at 4°C. Specimens were then washed thoroughly in PBST and mounted in Vectashield containing DAPI to label nuclei (Vector Laboratories). Labels for type I pro-collagen, gap junction protein CX43 and dendritic cell marker podoplanin (E11) were performed by indirect immunohistochemical procedures. In all cases, the primary antibody was replaced in control sections with PBST alone or with non-immune immunoglobulins (IgG), to reveal any non-specific binding. In all cases both PBST and IgG controls were negative. Type I pro-collagen was labeled using monoclonal antibody M38 recognizing the C-terminus of pro-collagen I in a wide range of species *except* mouse [5 μg/mL; (52); Developmental Studies Hybridoma Bank], CX43 using monoclonal antibody CXN-6 (8 μg/mL; Sigma) and E11 with goat anti-mouse podoplanin (E11) primary antibody (2.5 μg/mL; R&D Systems). Sections were blocked with 5% goat serum (those stained with antibodies M38 and CXN-6; Dako) or 5% rabbit serum (E11; Dako) for 30 min and then incubated with primary antibodies overnight at 4°C (E11) or 1 h at room temperature (M38, CXN-6). After extensive washing with PBST, M38 and CXN-6 sections were incubated with AlexaFlour594 or AlexaFluor488 goat anti-mouse conjugates, for 30 min at room temperature. All sections were thoroughly washed and mounted in Vectashield/DAPI. E11 sections were incubated with a horseradish peroxidase rabbit anti-goat conjugate (Vector Laboratories; 1:800 dilution) for 30 min, washed in PBST and label visualized using a nickel-enhanced diaminobenzidine peroxidase substrate kit (Vector Laboratories). Fluorescence specimens were examined with an Olympus BX61 epi-fluorescence microscope equipped with an F-view camera and AnalySISimage capture and analysis software, or using a Leica TCS SP2 AOBS confocal scanning laser microscope and using the Leica Confocal Software, and peroxidase labels with a Leica DMRB microscope with a Moticam 2000 camera. Confocal microscopy was carried out on samples using appropriate excitation and emission settings for simultaneous recording of DAPI and ethidium homodimer as previously outlined, Phalloidin-Atto488 [495 nm Ex_(max)_, 519 nm Em_(max)_], and Alexa 594 [590 nm Ex_(max)_, 617 nm Em_(max)_]. Specimens were optically sectioned using a x63 objective with an arbitrary zoom (surface and deep zone actin filament stain, and CX43 immunofluorescence). 5 μm (surface zone) or 0.5 μm (deep zone) step size z-stack optical sections through the specimen were reconstructed using Leica Confocal Software. Maximum intensity models were prepared showing detail of the surface zone or deep zone.

### Molecular analysis of osteocyte and osteoblast phenotype

Osteocyte and osteoblast phenotype was determined at the molecular level using reverse-transcriptase quantitative polymerase chain reaction (RT-qPCR). Ribonucleic acid (RNA) was extracted separately from surface and embedded cells of co-cultures grown in plastic plates by dispensing 1 mL Trizol (Invitrogen) onto the surface for 10 s to extract osteoblast RNA, and subsequently dissolving the underlying gel within a separate 1 mL Trizol aliquot to extract RNA from gel embedded cells. After RNA extraction according to manufacturer’s protocol and DNAse treatment (DNA-free, Ambion) RNA was re-precipitated with 3 M sodium acetate (pH 5.5, Ambion) and dissolved in molecular biology grade water (25 μL for surface cells, 50 μL for deep cells). RNA with A_260_/A_280_ and A_260_/A_230_ ratios of ≥1.8 was deemed good quality. RNA (1.7–253.3 ng for the surface zone; 108.8–1864.9 ng for the deep zone) was primed with random hexadeoxynucleotides (Promega, Southampton, UK) and reverse transcribed according to manufacturer’s instructions (Superscript III, Invitrogen). Gene expression of E11, osteocalcin (OCN), runt-related transcription factor 2 (Runx2), type I collagen (*Col1a1*), ALP, and simian vacuolating virus 40 (SV40) large T-antigen were analyzed by RT-qPCR [SYBR Green I master mix (Sigma), 2.5 mM MgCl_2_, 200 nM each primer, Stratagene MX3000P]. All targets except OCN used intron-spanning primers (Table 1) and no template controls were included for all reactions. Thermocycling included 1 cycle of 95°C for 3 min, 40 cycles of 95°C for 20 s, 60°C for 40 s, 72°C for 20 s, and 1 cycle of 72°C for 10 min. Specificity of RT-qPCR was confirmed by melting curve analysis (Stratagene MX3000P) with complementary deoxyribonucleic acid (cDNA) standard curves of 90–110% efficiency and with an *R*^2^ value of ≥0.99 accepted as valid.

**Table 1:** **Primer details**.

Gene	Primers (5′–3′)	Amplicon size (bp)
*GAPDH* (human) NM_002046.5	Fwd – GGT ATC GTG GAA GGA CTC ATG A	68
	Rev – GGC CAT CCA CAG TCT TCT G	
*HPRT1* (human) NM_000194.2	Fwd – GCA GAC TTT GCT TTC CTT GG	80
	Rev – GTG GGG TCC TTT TCA CCA G	
18S rRNA NR_003278.3	Fwd – GCA ATT ATT CCC CAT GAA CG	125
	Rev – GGC CTC ACT AAA CCA TCC AA	
E11 (human) NM_006474.4	Fwd – ACG GAG AAA GTG GAT GGA GA	186
	Rev – ACG ATG ATT GCA CCA ATG AA	
*RUNX2* (human) NM_ 001024630.3	Fwd – GTG GAC GAG GCA AGA GTT TC	107
	Rev – TTC CCG AGG TCC ATC TAC TG	
*COL1A1* (human) NM_000088.3	Fwd – CAG CCG CTT CAC CTA CAG C	83
	Rev – TTT TGT ATT CAA TCA CTG TCT TGC C	
OCN (human) NM_199173.4	Fwd – GGC AGC GAG GTA GTG AAG AG	73
	Rev – GAT CCG GGT AGG GGA CTG	
*Gapdh* (mouse) NM_ 001289726.1	Fwd – GAC GGC CGC ATC TTC TTG TGC A	114
	Rev – TGC AAA TGG CAG CCC TGG TGA C	
*Hprt* (mouse) NM_013556.2	Fwd – CGTGATTAGCGATGATGAACCAGGT	149
	Rev – CCATCTCCTTCATGACATCTCGAGC	
E11 (mouse) NM_010329.3	Fwd – AAG ATG GCT TGC CAG TAG TCA	118
	Rev – GGC GAG AAC CTT CCA GAA AT	
*Runx2* (mouse) NM_ 001146038.2	Fwd – GAC GAG GCA AGA GTT TCA CC	120
	Rev – GTC TGT GCC TTC TTG GTT CC	
*Col1a1* (mouse) NM_007742.3	Fwd – ACT GCC CTC CTG ACG CAT GG	140
	Rev – TCG CAC ACA GCC GTG CCA TT	
OCN (mouse) NM_007541.3	Fwd – CCGCCTACAAACGCATCTAT	153
	Rev – TTTTGGAGCTGCTGTGACAT	
ALP (mouse) NM_ 001287172.1	Fwd – GCTGGCCCTTGACCCCTCCA	132
	Rev – ATCCGGAGGGCCACCTCCAC	
SV40 YP_003708382.1	Fwd – AGGGGGAGGTGTGGGAGGTTTT	100
	Rev – TCAGGCCCCTCAGTCCTCAC	

Relative quantification of messenger ribonucleic acid (mRNA) expression by RT-qPCR was calculated using the 2^−ΔΔCT^ method (53). In all cases, RT-qPCR was carried out using three reference genes (RG), 18S rRNA, glyceraldehyde 3-phosphate dehydrogenase (*GAPDH*), and hypoxanthine–guanine phosphoribosyltransferase (HPRT1). The most stable RG was determined via NormFinder. Expression data for each gene in an experiment were transformed from log_10_ to linear scale using a standard curve and then loaded into the NormFinder Microsoft Excel Add-In^1^ (54). The most stable gene or combination of two genes, combined by calculating the geometric mean, was used for normalization and is clearly stated in the relevant figures. Target gene expression levels were expressed as relative expression units (REU), using the highest expressor within all samples as the calibrator sample.

### Mechanical loading

**Figure 2:**
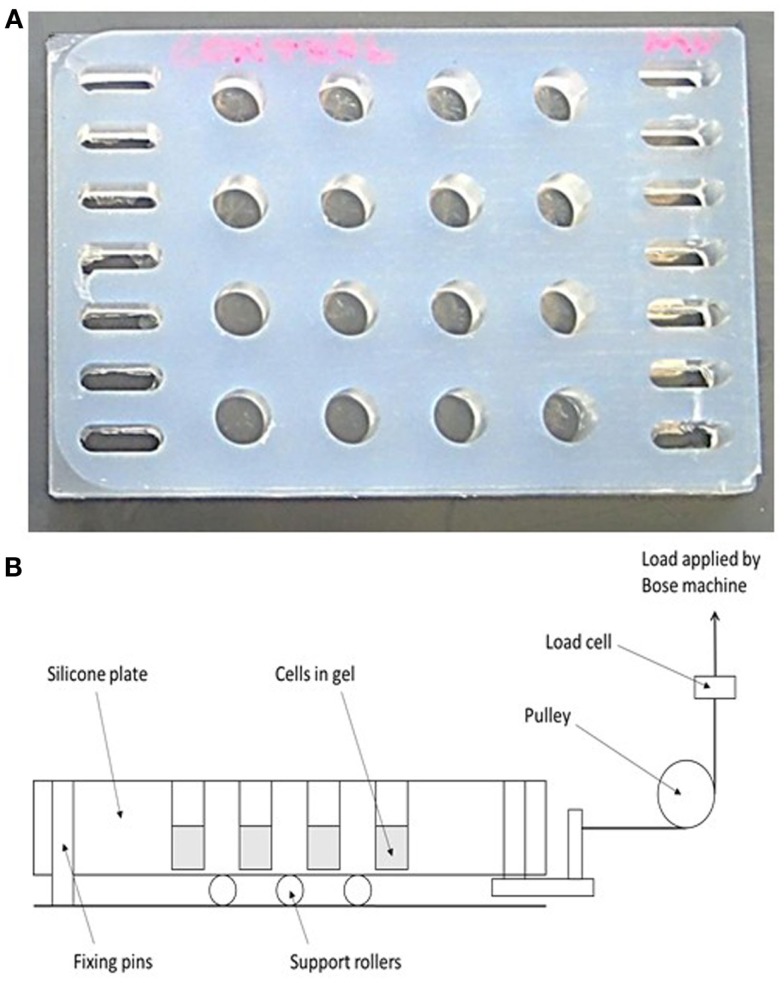
**A novel mechanical loading device**. **(A)** A 16-well rubber loading plate of the same dimensions (10 mm diameter) as a standard 48-well tissue culture plate but with a 150 μm thick base and with holes for its attachment to a BOSE loading instrument. **(B)** Schematic showing the operation of the loading device. The collagen gels are contained in the wells of the silicone plate, and the entire plate is stretched to apply a strain to the gels.

A 16-well loading plate (Figure 2A) was manufactured from solid silicone (Technovent TechSil 25)^2^ so that the wells of the plate were of the same dimensions (10 mm diameter) as a standard Greiner (Stonehouse, UK) 48-well tissue culture plate but with a 150 μm thick base. The spaces between the wells were filled with silicone and a series of holes were made on each side of the plate to accommodate hooks for attachment to a BOSE loading instrument. A Dantec Dynamics Digital Image Correlation (DIC) system was used to measure strain in the loading plate in order to calibrate the system. DIC compares two digital images of two different mechanical states of a particular object: a reference state and a deformed state. A previously applied speckle pattern (here applied using black face paint)^3^ follows the strain of the object, and so the displacement that occurs between both reference and deformed state can be measured by matching the speckle pattern in small regions of the image (55, 56). By using two cameras (Limess Messtechnik)^4^ and matching speckle patterns in each image, the position and displacement in 3D can be obtained, after calibrating the system using a grid of known dimensions to determine the position of the cameras. DIC validated strains of 4000–4500 με in the majority of the wells of the loading plate when a force of 2.5 N was applied.

For mechanical loading the silicone plate was attached to a BOSE ElectroForce^®^ 3200 (Kent, UK) loading instrument by a custom-made device (Figure 2B) in order to stretch the plate from one end causing cyclic compression in all wells. A 250 N load cell was used to apply a loading regime of 5 min, 10 Hz, 2.5 N to 3D osteocyte mono-cultures. Loading was controlled using WinTest^®^ Software 4.1 with TuneIQ control optimization (BOSE).

For loading, 3D osteocyte mono-cultures were prepared and cultured in the silicone plate in 800 μl of DMEM GlutaMAX™ supplemented with 100 U/ml penicillin, 100 μg/ml streptomycin, and 5% DFBS incubated at 37°C in 5% CO2/95% air atmosphere for 24, 48, or 72 h without changing culture medium prior to load, or 7 days where culture medium was changed every 2–3 days and prior to loading. 3D co-cultures were prepared and cultured in the silicone plate as described previously for plastic plates and cultured for 7 days prior to load, changing culture medium every 2–3 days and immediately prior to loading.

Prostaglandin E_2_ release was measured at 0, 0.5, 1, 3, 6, 12, and 24 h post-load using an Enzo Life Sciences PGE_2_ kit (Exeter, UK) and following manufacturer’s instructions. Experimental samples were diluted 1:64 (gels set up 24 h before loading), 1:16 (gels set up 48 and 72 h before loading), or 1:40 (gels set up 7 days before loading) in order to fit within the standard curve of the assay. All samples were within the standard curve range. The sensitivity of the assay is 8.26 μg as stated by the manufacturer. Absorbance was recorded using a BMG Labtech FLUOstar Optima plate reader (Bucks, UK) and using the Optima Software for FLUOstar V2.00 R3 (BMG Labtech). PGE_2_ concentration of the experimental samples was determined according to the standard curve. Data was normalized to total cell number by lysing cultures and performing LDH assay (CytoTox 96^®^ Non-Radioactive Cytotoxicity Assay, Promega, Southampton, UK).

PINP (type I pro-collagen) synthesis was measured at day 1 and 5 post-load using a Rat/Mouse PINP Enzyme Immunoassay (EIA) kit (Immunodiagnostic systems, Tyne & Wear, UK) following the manufacturer’s instructions. All samples were within the standard curve range. The sensitivity of the assay was 0.7 ng/ml as stated by the manufacturer. Data were normalized to total DNA content (extracted using TRIzol^®^ reagent and quantified after precipitation using a Quant-iT™ dsDNA High-Sensitivity Assay Kit, both following the manufacturer’s instructions). The sensitivity of the DNA assay was 0.5 ng/μl.

### Statistics

Data are expressed as the mean ± Standard Error of the Mean (SEM). Residuals were tested for normality (Anderson–Darling) and equal variance (Bartlett’s and Levene’s tests) and transformed if necessary, before applying analysis of variance (ANOVA) and *post hoc* Fisher’s or Tukey’s tests or General Linear Model (GLM) for crossed factors with pairwise comparisons where *P* < 0.05 were recorded. Data were deemed to be significantly different when *P* < 0.05. 3D cultures prepared in individual wells within a plate, cultured for 1–12 days and loaded/treated were considered independent replicates. Group of independent replicates (3D cultures) prepared, cultured, and loaded/treated in separate plates on separate occasions were considered independent experiments.

## Results

### Osteocyte and osteoblast viability in 3D co-cultures

**Figure 3:**
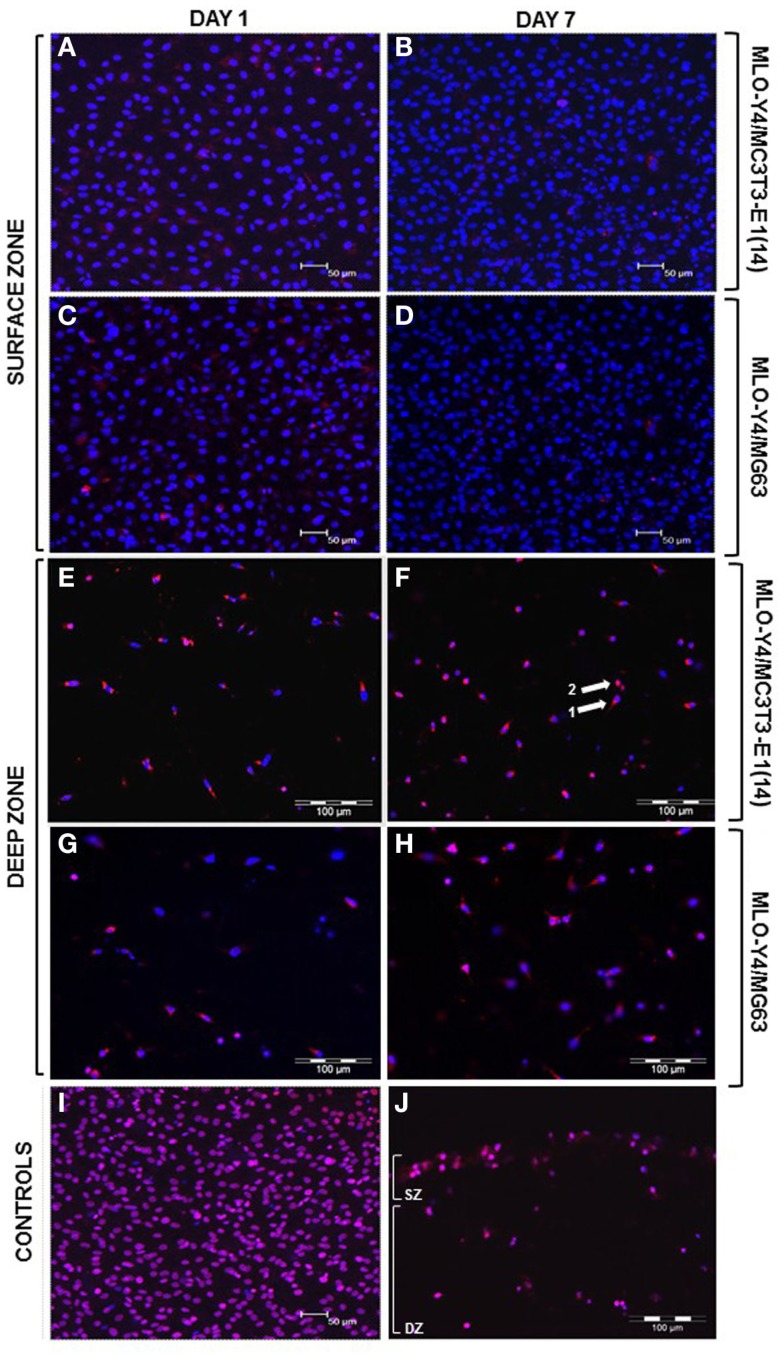
**Viability of the surface and deep zone cells in 3D co-cultures (red: ethidium homodimer; blue: DAPI, purple: combination of both dyes)**. Confocal microscope images of an arbitrary field of view showing only live (blue nuclei) surface cells at day 1 **(A,C)** and at day 7 **(B,D)** of both MLO-Y4/MC3T3-E1(14) **(A,B)** and MLO-Y4/MG63 **(C,D)** co-cultures, respectively. Some live osteoblasts show red staining in their cytoplasm but not their nucleus at both time-points. Fluorescence microscope images of an arbitrary field of view showing a mixture of live and dead embedded cells at day 1 **(E,G)** and at day 7 **(F,H)** in both MLO-Y4/MC3T3-E1(14) **(E,F)** and MLO-Y4/MG63 **(G,H)** co-cultures, respectively. Some live osteocytes also show red staining [arrow 1 in **(F)**] in the cytoplasm but not their nucleus, whereas, dead cells are red throughout [arrow 2, **(F)**]. Confocal microscope image of an arbitrary field of view from a freeze-thaw control where all surface cells are dead **(I)**. Fluorescence microscope image of an arbitrary field of view from a freeze-thaw control where all surface and embedded cells are dead **(J)** (SZ: surface zone, DZ: deep zone). For the osteoblasts, images were taken at the level of the surface zone and are representative of three independent experiments, *n* = 3 per experiment, five arbitrary fields of view per replicate. Scale bars: 50 μm **(A–D, I)**. For the osteocytes, images were taken from 3D co-culture transverse cryosections and are representative of two or three independent experiments, *n* = 3 per experiment, 5 sections per replicate, 10 arbitrary fields of view per section [scale bars: 100 μm **(E–H, J)**].

Confocal images of the surface zone across five arbitrary fields of view were taken for all replicates of both MLO-Y4/MC3T3-E1(14) (three independent experiments of *n* = 3) or MLO-Y4/MG63 (two independent experiments of *n* = 3) 3D co-cultures grown in plastic plates. At both day 1 and day 7, the viability of MC3T3-E1(14) (Figures 3A,B, respectively) or MG63 (Figures 3C,D, respectively) osteoblast-like cells was 100%. Freeze-thaw controls showed 100% death of the surface zone of the model (Figure 3I).

Ten arbitrary fields of view from five random transverse cryosections from all replicates were used for quantification of MLO-Y4 cell death as a proportion of total MLO-Y4 number in both MLO-Y4/MC3T3-E1(14) (three independent experiments of *n* = 3) or MLO-Y4/MG63 (one independent experiment of *n* = 3 for day 1; two independent experiments of *n* = 3 for day 7) 3D co-cultures. At day 1 and day 7, a mixture of live and dead MLO-Y4 cells was observed for both MLO-Y4/MC3T3-E1(14) (Figures 3E,F, respectively) and MLO-Y4/MG63 (Figures 3G,H, respectively) co-cultures. Live osteocytes had a blue nucleus and dendritic morphology, whereas, a purple nucleus and rounded morphology was observed for dead osteocytes. Some live MLO-Y4 cells had red staining in their cytoplasm but not their nucleus (arrow 1, Figure 3F). Freeze-thaw controls showed 100% osteocyte death (Figure 3J).

**Figure 4:**
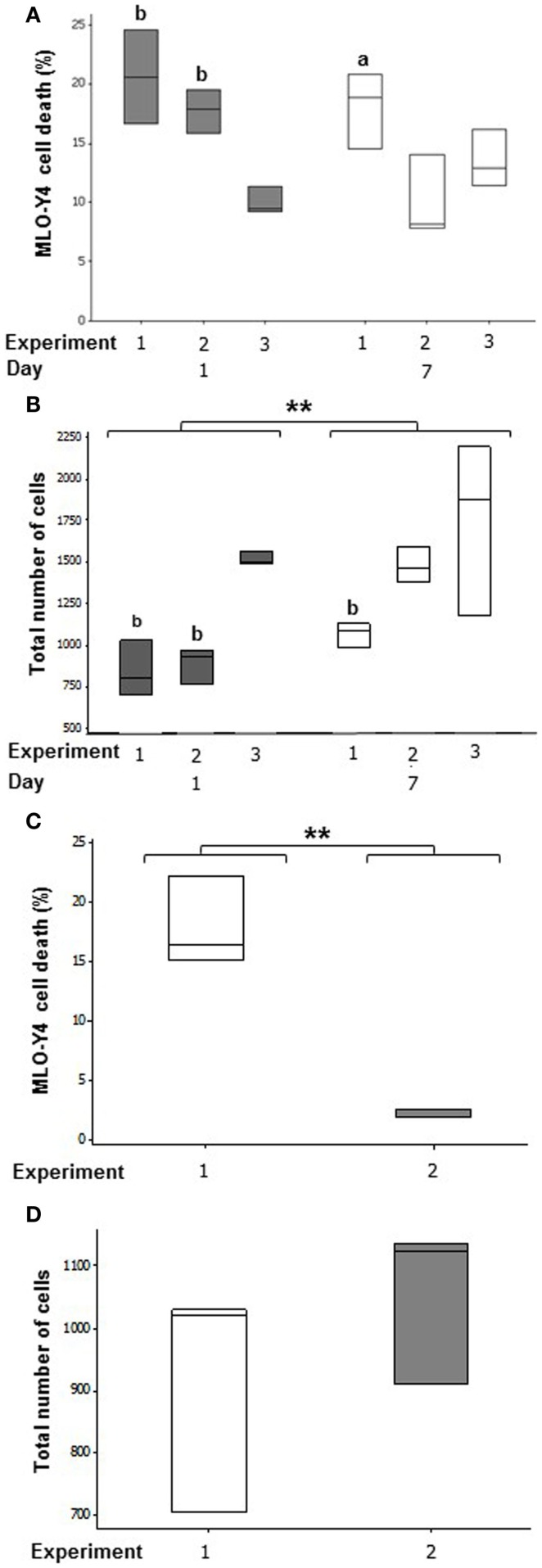
**Quantification of cell number and cell death in the deep zone of 3D co-cultures**. Boxplots of percentage cell death as a proportion of total number of cells at day 1 and day 7 in MLO-Y4/MC3T3-E1(14) **(A)** and MLO-Y4/MG63 **(C)** co- cultures. Boxplots showing total cell number counted for each replicate experiment at day 1 and day 7 in each independent experiment for MLO-Y4/MC3T3-E1(14) **(B)** and MLO-Y4/MG63 **(D)**. For total cell number, significant differences obtained by GLM of log_10_ data between day 1 and day 7 denoted by **P < *0.01*. Significant differences from pairwise comparisons, within each day, between independent experiments are shown by “a,” with respect to experiment 2; and “b,” with respect to experiment 3.

In MLO-Y4/MC3T3-E1(14) co-cultures, an average of 16.13 ± 3.16% osteocyte cell death was observed at day 1 and 13.85 ± 2.35% at day 7 (Figure 4A). Mean MLO-Y4 cell death within 3D co-cultures did not differ between day 1 and day 7, however, MLO-Y4 cell death varied significantly between independent experiments (GLM, *P* = 0.002) with a significant interaction between day and experiment (GLM, *P* = 0.018). MLO-Y4 cell death at day 1 was significantly reduced in experiment 3 (10.04 ± 1.14%) compared with experiment 1 (20.62 ± 2.28%, *P* = 0.007) and experiment 2 (17.32 ± 1.43%, *P* = 0.004) whereas, MLO-Y4 cell death at day 7 differed significantly between experiment 1 (18.08 ± 1.86%) and experiment 2 (9.35 ± 1.39%) (*P* = 0.041).

Total cell number increased between day 1 and day 7 (GLM, *P* = *0.003* of log_10_ data), and varied between replicate experiments (GLM, *P* < 0.001 of log_10_ data) in MLO-Y4/MC3T3-E1(14) co-cultures. At day 1, total cell number was 2-fold higher in experiment 3 when compared to experiment 1 (GLM, *P* = 0.009 of log_10_ data), and 1.5-fold higher when compared to experiment 2 (GLM, *P* = 0.019 of log_10_ data). At day 7, total cell number was 1.7-fold higher in experiment 3 when compared to experiment 1 (GLM, *P* = *0.049* of log_10_ data) (Figure 4B).

After 7 days of co-culture, the mean percentage of dead MLO-Y4 cells in MLO-Y4/MG63 co-cultures was 9.98 ± 3.66% across both independent experiments. However, there was a significant difference observed between mean percentage death of experiments 1 (17.88 ± 2.16%) and 2 (2.08 ± 0.22%) (One-way ANOVA, *P* = 0.002) (Figure 4C). There was no significant difference observed in total MLO-Y4 cell number at day 7 between experiments 1 and 2 in MLO-Y4/MG63 co-cultures (Figure 4D).

### Osteocytes and osteoblasts assume appropriate morphology in co-cultures

**Figure 5:**
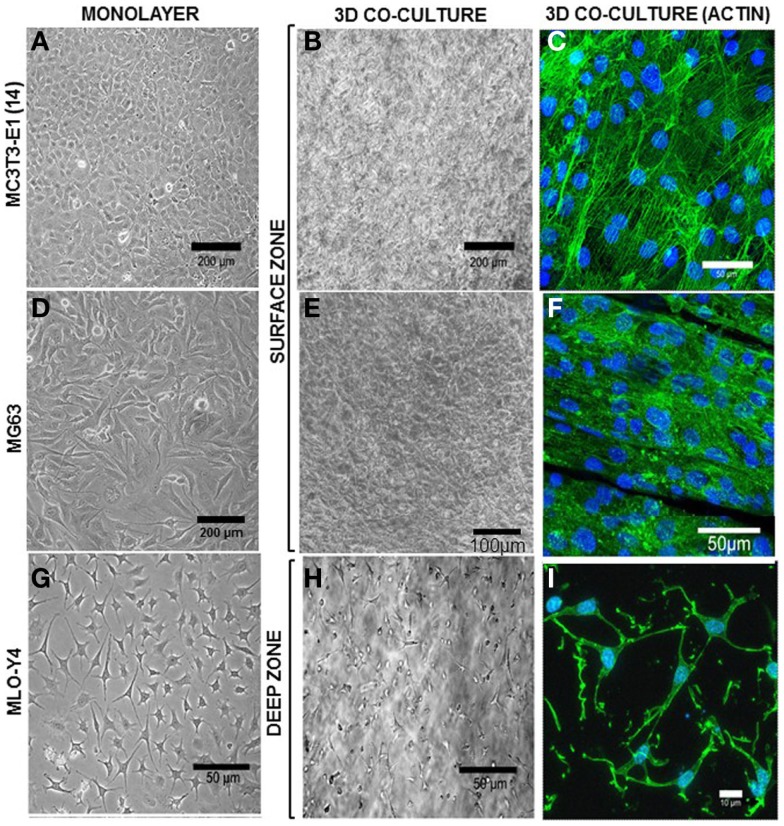
**Morphology of surface and deep zone cells in 3D co-cultures**. Inverse light microscope images of MC3T3-E1(14) **(A)**, MG63 **(D)**, and MLO-Y4 **(G)** cells in monolayer. MC3T3-E1(14) and MG63 osteoblast-like cells present typical osteoblastic morphologies, mainly ovoid or pyriform, and MLO-Y4 osteocyte-like cells have a typical dendritic morphology. Inverse light microscope images taken from the surface zone **(B,E)** of the 3D model where a confluent monolayer of surface cells is observed, and halfway through the depth of a 3D co-culture **(H)** showing embedded cells with a similar dendritic morphology to that seen in MLO-Y4 monolayer cultures. Confocal microscope focusing directly onto the surface zone of a 3D co-culture at day 7 **(C,F)**, where surface cells were stained to reveal actin filaments (Phalloidin-Atto488) and cell nuclei (DAPI). The image shows a pavement-like monolayer, with individual cells containing well-developed stress-fibers, and maintaining an osteoblastic morphology. Confocal microscope image stack of a 3D co-culture transverse cryosection at day 7 **(I)** where embedded cells were stained to reveal actin filaments (Phalloidin-Atto488) and cell nuclei (DAPI), showing a dendritic morphology and connections between neighboring cells. Images are arbitrary fields of view representative of three independent experiments, *n* = 3 per experiment.

MLO-Y4 osteocyte-like cells embedded within a 3D type I collagen gel overlaid with either MC3T3-E1(14) or MG63 osteoblasts grown in plastic plates revealed a single osteoblast surface cell layer and dendritic MLO-Y4 cells embedded throughout the depth of the type I collagen gel (Figure 5).

In MLO-Y4/MC3T3-E1(14) co-cultures, MC3T3-E1(14) cells had a similar ovoid or pyriform morphology to those in monolayer cultures (Figure 5A). The MC3T3-E1(14) osteoblasts formed a thin pavement-like single cell layer on top of the 3D co-cultures, which were difficult to image under the inverse light microscope (Figure 5B). However, actin filament labeling (Figure 5C) revealed MC3T3-E1(14) osteoblast morphology more clearly, with stress-fibers throughout their cell bodies. With similar morphology to MG63 monolayer cultures (Figure 5D), MG63 cells formed a pavement-like single cell layer in MLO-Y4/MG63 co-cultures under both light microscopy (Figure 5E) and immunofluorescence, when filamentous actin was labeled (Figure 5F). MLO-Y4 dendritic morphology observed in monolayer cultures (Figure 5G) was similar in co-cultures (Figure 5H), although projections extended in three dimensions. Contacts between neighboring osteocytes were revealed by actin filament staining (Figure 5I).

### Osteocytes and osteoblasts display appropriate phenotype in 3D co-cultures

mRNA expression was assessed in both surface and deep zones of day 7 MLO-Y4/MC3T3-E1(14) 3D collagen co-cultures grown in plastic plates by relative RT-qPCR using primers against osteoblast and osteocyte phenotypic markers. Data were expressed in REU and normalized to *Gapdh*, which was ranked as the most stable reference gene (NormFinder stability value = 0.398, intergroup variation = 0.376, and intragroup variation = 0.012). Data were analyzed from three independent experiments, each with three replicates for the surface zone, and four replicates for the deep zone, for all genes except *Col1a1* (two independent experiments).

**Figure 6:**
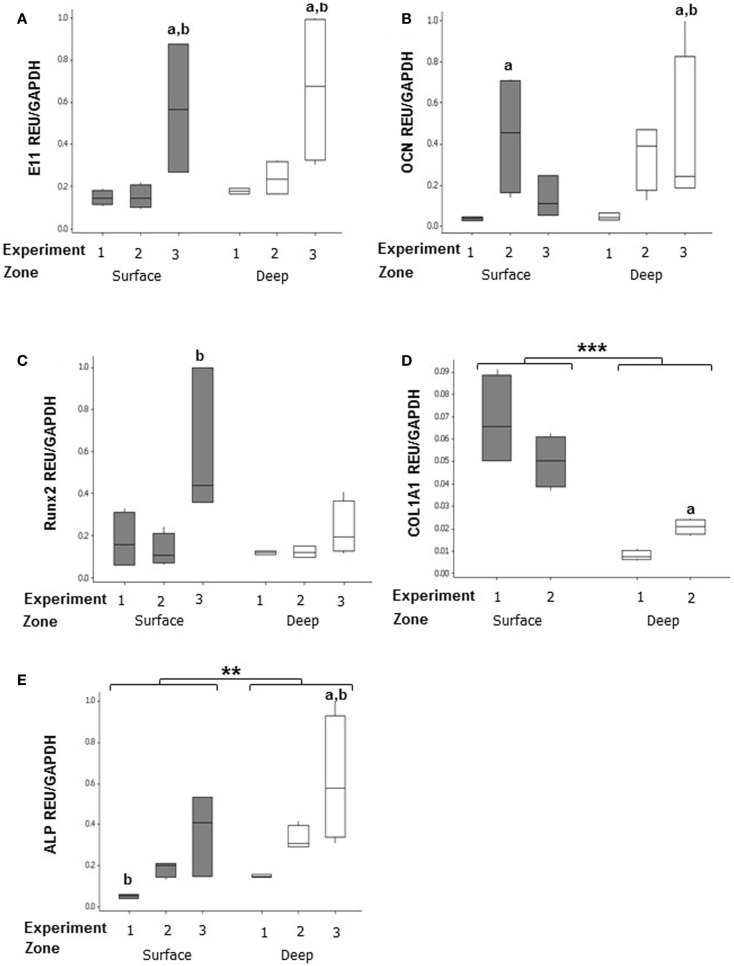
**Gene expression of cellular markers in surface and deep zone cells in MLO-Y4/MC3T3-E1(14) 3D co-cultures**. Quantification of gene expression in the 3D co-culture after 7 days by relative RT-qPCR, boxplots of E11 **(A)**, OCN **(B)**, RUNX2 **(C)**, *Col1a1* **(D)**, and ALP **(E)** expressed as REU and normalized to *Gapdh* expression. Significant differences obtained by GLM of log_10_ data (E11, *Col1a1*, and OCN) or ranked data (ALP and Runx2) between surface and deep zones denoted by **P < *0.01*, ***P < *0.0001*. Significant differences from pairwise comparisons, within each zone, between independent experiments denoted by “a,” with respect to experiment 2; and “b,” with respect to experiment 3. Values derived from two (*Col1a1*) or three (all others) independent experiments, *n* = 3 for surface and four for deep zones.

In MLO-Y4/MC3T3-E1(14) co-cultures, no significant difference in expression was detected between zones of the model for E11 (surface zone, 0.264 ± 0.072 REU; deep zone, 0.361 ± 0.087 REU) (Figure 6A), OCN (surface zone, 0.212 ± 0.076 REU; deep zone, 0.269 ± 0.080 REU) (Figure 6B), and Runx2 (surface zone, 0.275 ± 0.083 REU; deep zone, 0.157 ± 0.025) (Figure 6C). However, the surface zone of the model showed 6-fold increases in expression of *Col1a1* compared to the deep zone (0.168 ± 0.085 vs. 0.028 ± 0.007 REU, GLM, *P* < 0.001 of log_10_ data) (Figure 6D). In contrast, the deep zone of the 3D co-culture showed 2-fold increases in ALP expression over the surface zone (0.366 ± 0.075 vs. 0.185 ± 0.047 REU, GLM, *P* = 0.001 of ranked data) (Figure 6E). Whilst REU of all genes varied significantly between replicate experiments (GLM, E11, OCN, and *Col1a1*, *P* < 0.001 of log_10_ data; Runx2 *P* = 0.013 of ranked data; ALP *P* < 0.001 of ranked data; *P* < 0.05 for all pairwise comparisons) the trend in terms of surface compared with deep REUs within each experiment was consistent. Consistent with this, RT-PCR of MLO-Y4/MG63 co-cultures, revealed surface osteoblasts and embedded osteocytes expressed E11, OCN, Runx2, and *COL1A1* mRNA (data not shown, three independent experiments of *n* = 3 for both surface and deep zones). Quantification of mRNA expression could not be compared between surface MG63 and embedded MLO-Y4 cells as the respective human and mouse cDNA sequences are not sufficiently homologous to use the same primers.

**Figure 7:**
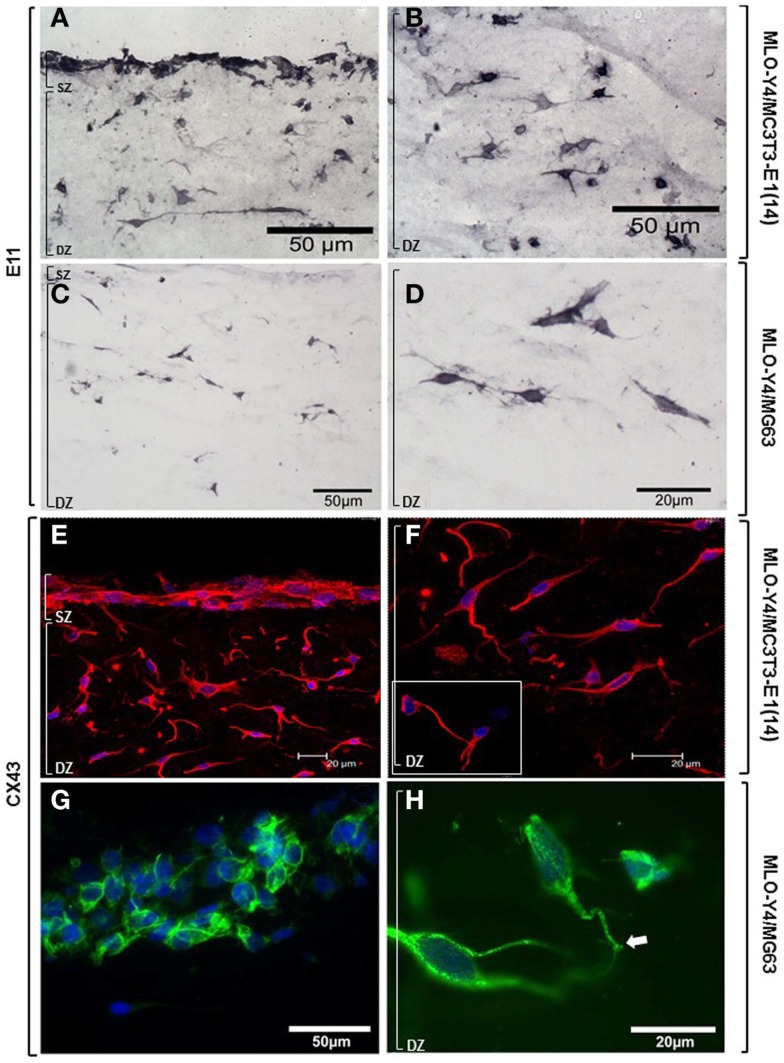
**Protein expression of cellular markers in surface (SZ) and deep (DZ) zone cells of the 3D co-culture systems**. Brightfield photomicrographs showing immunostaining for the dendricity marker E11 in both surface and embedded cells **(A)** and showing E11 immunostaining in the osteocytes highlighting their morphology **(B)**, in MLO-Y4/MC3T3- E1(14) 3D co-cultures. Light microscope images revealing immunostaining for the dendricity marker E11 in embedded cells **(C,D)** but not in surface cells **(C)**, in MLO-Y4/MG63 co-cultures. Confocal microscope images showing CX43 (Dylight594) immunolabelling and cell nuclei stain (DAPI) in surface and deep zone cells **(E)** of MLO-Y4/MC3T3-E1(14) co-cultures. Image reveals abundant quantities of CX43 present in the cytoplasm and cell membranes of both cell types, around the nucleus of the embedded cells **(F)**, and connections between neighboring cells [**(F)**, inset] (inset scale bar: 10 μm). Fluorescent photomicrographs of surface **(G)** and deep **(H)** zone cells of MLO-Y4/MG63 co-cultures labeled for CX43 (green) and counterstained with DAPI (blue) reveals that the surface cell layer, in this case several cells thick, intensely labels for CX43 along cell–cell interfaces **(G)**. High magnification of embedded cells within the same co-culture gel reveals extensive punctate labeling within the cytoplasm and at the cell surface, including at interfaces between cell processes (arrow) **(H)**. Images are arbitrary fields of view taken from 3D co-culture transverse cryosections representative of two or three independent experiments, *n* = 3 per experiment. In all cases, controls performed by omitting or substituting the primary antibody, showed no labeling.

Osteoblasts and osteocytes in MLO-Y4/MC3T3-E1(14) co-cultures showed strong, uniform immunolabelling for the dendricity marker E11 (Figures 7A,B). Intense E11 immunolabelling was also observed in embedded MLO-Y4 cells within the MLO-Y4/MG63 co-cultures, but not in surface MG63 cells (Figures 7C,D). In both 3D co-culture systems abundant CX43 immunostaining was observed in the cell membrane and cytoplasm of osteoblasts and in osteocytes along their processes, as well as within the cytoplasm, around the nucleus (Figures 7E–G) and in contacts between cells (Figures 7F inset; 7H). Immunohistochemistry images are representative of day 7, 3D co-cultures from three independent experiments where *n* = 3 [MLO-Y4/MC3T3-E1(14)], or two independent experiments where *n* = 3 (MLO-Y4/MG63). Four to six cryosections from all replicates were observed. PBST and IgG controls were negative.

### Cell migration in co-cultures

**Figure 8:**
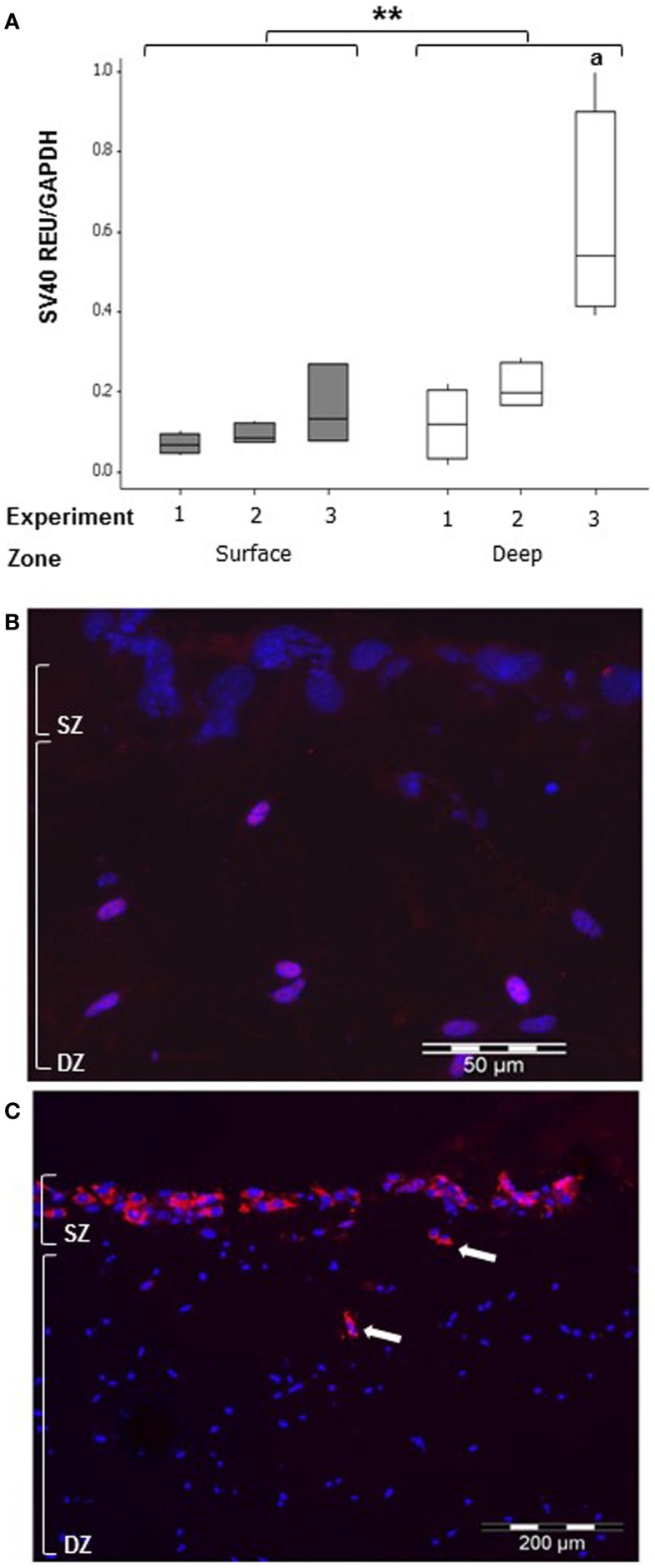
**Co-cultures retain zone separation for up to 7 days (SZ: surface zone; DZ: deep zone)**. Boxplot showing quantification of SV40 large T-antigen gene expression in the MLO-Y4/MC3T3-E1(14) co-culture after 7 days by relative RT-qPCR **(A)** expressed as REU and normalized to *Gapdh* expression. Significant differences obtained by GLM of log_10_ data between surface and deep zones denoted by **P < 0.01. Significant differences from pairwise comparisons, within each zone, between independent experiments denoted by “a” with respect to experiment 1 (three independent experiments, *n* = 3 for surface and 4 for deep zones). Fluorescent photomicrograph of transverse cryosection from day 7 MLO-Y4/MC3T3-E1(14) co-culture shows immunolabelling for SV40 large T-antigen (red) and cell nuclei stain (blue) in osteocytes only, represented by the purple color (red and blue co-localization) **(B)**. However, no SV40 large T-antigen immunostaining in the osteoblasts was present (three independent experiments, *n* = 3). SZ, surface zone; DZ, deep zone. Fluorescent photomicrograph of transverse cryosection from BMP-2 treated MLO-Y4/MG63 co-cultures at day 5 **(C)** revealed presence of type I pro-collagen in the upper layer of cells and in cells up to 100 μm beneath the surface, which are all MG63 cells since M38 antibody does not recognize mouse type I pro-collagen (two independent experiments,*n* = 3).

To detect whether MLO-Y4 cells moved to the surface zone, expression of the SV40 large T-antigen (only expressed by MLO-Y4 cells) was determined in MLO-Y4/MC3T3-E1(14) co-cultures grown in plastic plates (Figure 8). Whilst low levels of SV40 large T-antigen mRNA expression were detected in the surface zone (Figure 8A), SV40 large T-antigen immunolabelling was completely absent from the surface zone of the model (Figure 8B).

Osteoblast migration from surface to deep zone could be tracked in MLO-Y4/MG63 co-cultures, using a type I pro-collagen antibody that only detects human (i.e., MG63-derived) pro-collagen and not that expressed by mouse. Immunolocalization revealed that MG63 cells synthesizing human type I pro-collagen, whilst abundant in the upper layer of cells were also occasionally observed in cells up to 100 μm beneath the surface zone (Figure 8C).

### BMP-2 treatment regulates MG63 expression of type I collagen in co-cultures

**Figure 9:**
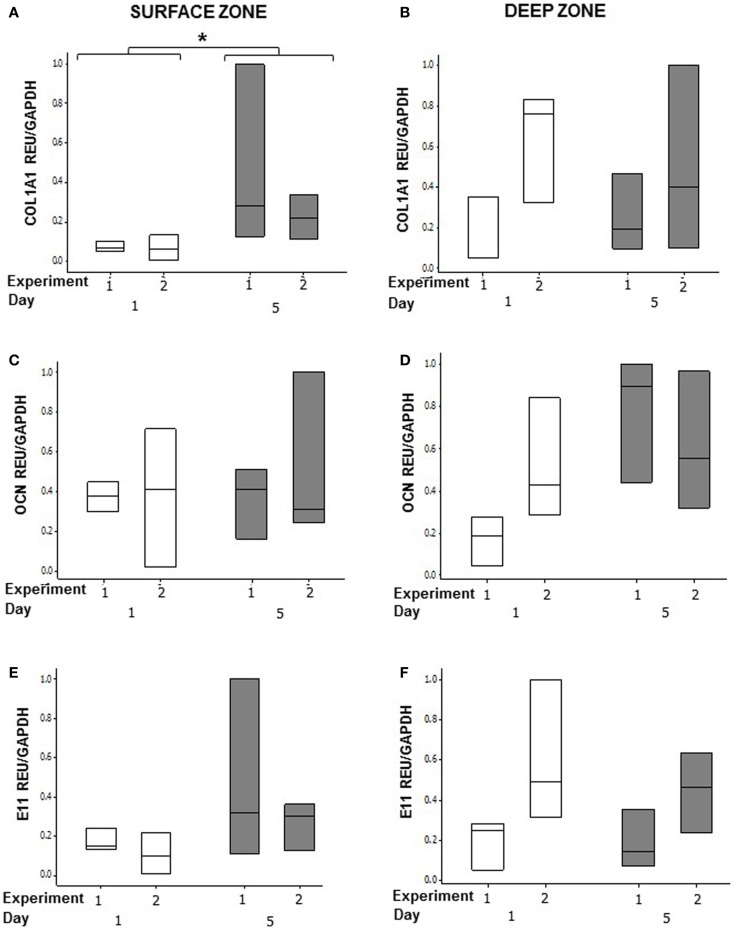
**Effects of BMP-2 treatment on osteoblast and osteocyte phenotype in MLO-Y4/MG63 3D co-cultures**. Quantification of gene expression in surface and deep zone cells of 3D co-cultures at day 1 and 5 after BMP-2 treatment by relative RT-qPCR, boxplots of *COL1A1* **(A,B)**, OCN **(C,D)**, and E11 **(E,F)** expressed as REU and normalized to *GAPDH* expression. Significant differences obtained by GLM of log_10_ data denoted by *P < 0.05. Data are from two independent experiments of *n* = 3 for surface and deep zones.

In order to determine whether osteoblasts in co-cultures could respond to an osteogenic signal, we stimulated the MLO-Y4/MG63 co-cultures grown in plastic plates with BMP-2 (Figure 9). We used the mouse/human model so that we could discriminate between MLO-Y4-derived and MG63-derived type I collagen expression. BMP-2 treatment significantly increased MG63 *COL1A1* mRNA expression at day 5 compared to day 1 (Figure 9A) (GLM of log_10_ data, *P* = 0.03, two independent experiments of *n* = 3). However, BMP-2 treatment had no effect on MLO-Y4 *Col1a1* (Figure 9B), MG63 or MLO-Y4 OCN (Figures 9C,D), and E11 (Figures 9E,F) mRNA expression.

**Figure 10:**
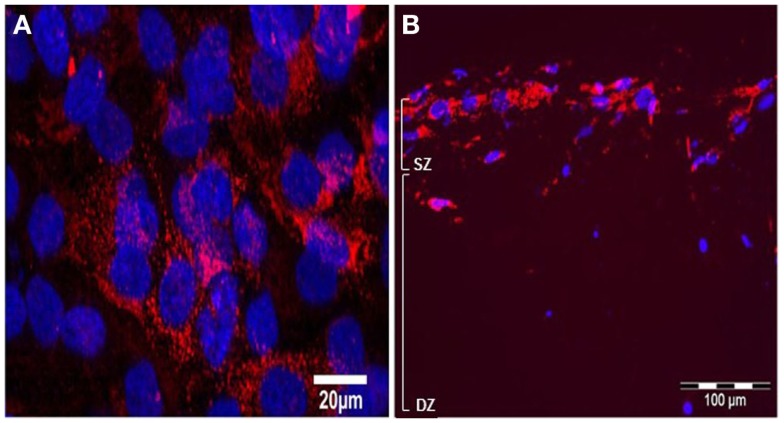
**Effects of BMP-2 treatment on type I pro-collagen synthesis in co-cultures of MLO-Y4 and MG63 cells (type I pro-collagen: red; DAPI: blue)**. Confocal microscope image of the surface cells from an untreated 3D co-culture revealed presence of particulate type I pro-collagen in all surface cells **(A)**. Fluorescence microscope images of transverse cryosections from BMP-2 treated 3D co-cultures at day 5 **(B)** revealed presence of type I pro-collagen in the upper layer of cells and in cells up to 100 μm beneath the surface, which are all MG63 cells since M38 antibody does not recognize mouse type I pro-collagen.

Immunolabelling with monoclonal antibody M38 that recognizes the C-terminus of human type I pro-collagen (an epitope not present in the collagen used to make the gel) revealed that type I pro-collagen is abundant in surface MG63 cells after 5 days BMP-2 treatment (Figures 10A,B).

### Embedded MLO-Y4 cells release PGE_2_ in response to mechanical loading

**Figure 11:**
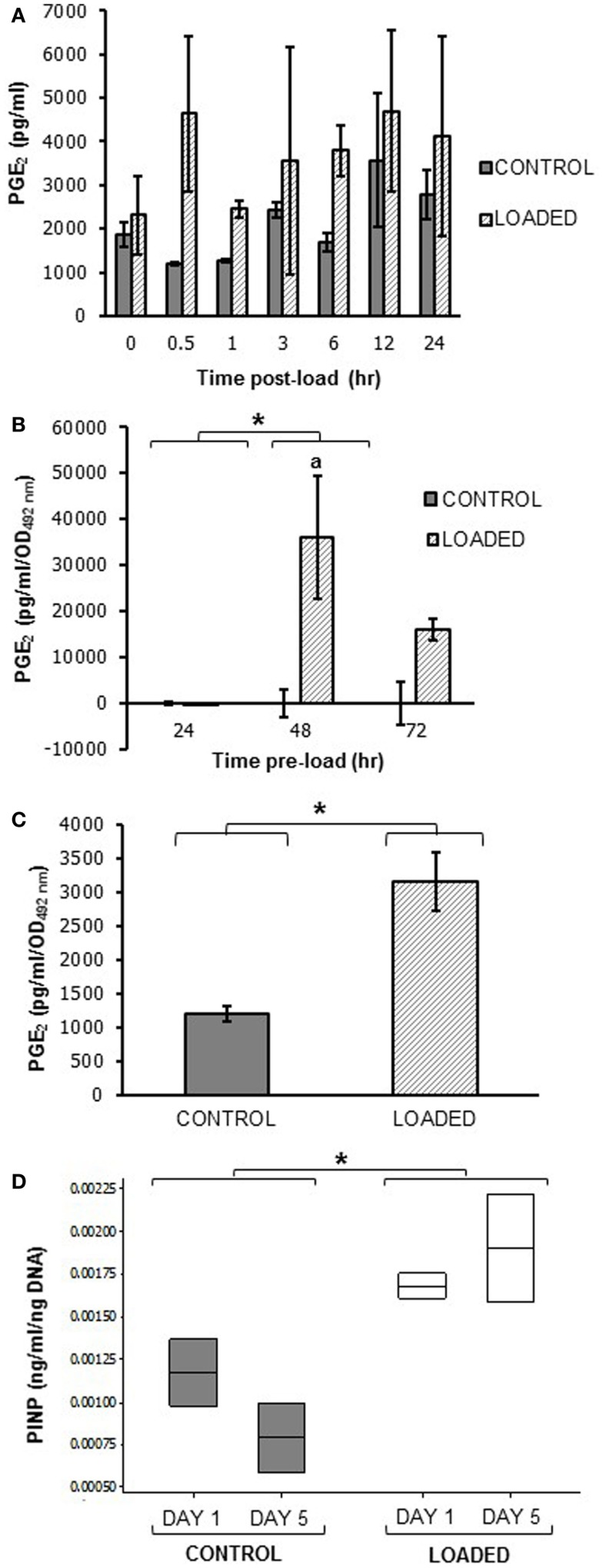
**Prostaglandin E_2_ and PINP release in mechanically loaded (5 min, 10 Hz, 2.5 N) 3D cultures by ELISA**. Graphs showing PGE_2_ release from 3D osteocyte mono-cultures in a pilot experiment of 24 h cultures **(A)**, categorized by time of culture **(B)**, and 7 days cultures **(C)** at 0.5 h post-load unless other time-points are indicated. Data were normalized to the absorbance (OD_492_ nm) of LDH lysates (cell number) **(B,C)**. **(D)** Boxplot of PINP release from control and loaded MLO-Y4/MC3T3-E1(14) 3D co-cultures cultures at day 1 and day 5 post-load, normalized to total DNA. *P < 0.05 as obtained by GLM, GLM of ranked data **(B)** or one-way ANOVA **(C,D)**. Significant differences as obtained by GLM pairwise comparisons denoted by “a” with respect to 24 h loaded cultures **(B)**. Data presented are from **(A)** one independent experiment, *n* = 2 or 3; **(B)** one (48–72 h cultures) or two (24 h cultures) independent experiments, *n* = 3; **(C,D)** one independent experiment, *n* = 2 or 3.

A pilot experiment to determine whether mechanical loading induced PGE_2_ release in MLO-Y4 cells in 3D gels in the silicone plate, revealed that load (5 min, 10 Hz, 2.5 N) increased PGE_2_ release between 0.5 and 24 h. Mean PGE_2_ release was increased approximately 4-fold at 0.5 h post-load (control 1206.55 ± 37.32 pg/ml; loaded 4632.91 ± 1773.78 pg/ml; *n* = 2 at each time) (Figure 11A).

To determine whether load-induced PGE_2_ release was affected by MLO-Y4 culture time in 3D gels prior to loading, MLO-Y4 cells were pre-cultured in gels for 24, 48, or 72 h and PGE_2_ measured 0.5 h after loading as before. After normalizing to cell number, PGE_2_ was not detectable in loaded or control 3D MLO-Y4 mono-cultures pre-cultured for 24 h, whereas mean PGE_2_ was increased in loaded osteocytes pre-cultured for 48 h and for 72 h compared with their respective unloaded controls (Figure 11B, *n* = 3 per pre-culture time). When MLO-Y4 cells were pre-cultured for 7 days prior to mechanical loading, mean PGE_2_ release, normalized to cell number, was also increased 0.5 h post-load [control 1195.40 ± 109.72 pg/ml/OD_492_ nm, loaded 3152.26 ± 435.20 pg/ml/OD_492_ nm; *n* = 2 or 3 (Figure 11C)].

To determine whether mechanical loading could induce type I pro-collagen synthesis a pilot experiment assessed PINP synthesis 1 and 5 days post-load. This was carried out in 3D MLO-Y4/MC3T3-E1(14) co-cultures grown in the silicone plate pre-cultured for 7 days prior to load. Our preliminary findings reveal mean PINP release was increased in loaded 3D co-cultures when compared to control cultures at day 1 or 5 (Figure 11D, *n* = 2 control and loaded).

## Discussion

This paper describes methodology for a novel *in vitro* 3D osteocyte–osteoblast co-culture model, which can be used to assess how osteocytes regulate osteoblasts in response to mechanical load. The model has been morphologically and phenotypically characterized, and methodology optimized based on responses to mechanical load. The model is a two-phase culture system where osteocytes are embedded within collagen gels and cultured overnight before osteoblasts were added to the surface of the gel. In this model, cells were viable, expressed appropriate phenotypic markers and contacted neighboring cells. A 16-well silicone plate was developed to enable application of physiological and osteogenic forces within each gel. Our preliminary findings indicate that these 3D cultures increase PGE_2_ synthesis and PINP release in response to mechanical loading.

### Osteocyte and osteoblast viability in 3D co-cultures

In the 3D co-cultures, both MC3T3-E1(14) and MG63 surface osteoblasts were 100% viable. It is likely that osteoblasts on the surface of the co-culture behave like a monolayer of cells, with dead osteoblasts detaching from the top of the collagen gel and being replaced by new osteoblasts to maintain the single cell layer.

In the 3D co-cultures, embedded osteocytes displayed 16% death after 1 day of culture [MLO-Y4/MC3T3-E1(14)] and 10–14% death at day 7 (both co-culture systems). Osteocyte death is common in normal human bone (57) and increases from <1% at birth up to 75% by 80 years old (58–60). If there is a linear relationship between age and osteocyte death, 20% osteocyte death would occur in humans in their early 20s, consistent with osteocyte viability observed in both 3D models. Cells undergoing cell death are usually destroyed by neighboring or phagocytic cells (61), but dead osteocytes, embedded within a mineralized matrix are inaccessible, and can be detected within their lacunae *in vivo* (62, 63). In the 3D co-culture a similar percentage osteocyte cell death was observed at day 1 and day 7, which may reflect dead osteocyte retention within the matrix, but this remains to be determined.

### Osteocyte and osteoblast morphology in 3D co-cultures

In the 3D co-culture model, both MC3T3-E1(14) and MG63 cells displayed a range of osteoblastic, ovoid, and pyriform morphologies, when maintained for 7 days. They formed a pavement-like monolayer on top of the 3D culture with well-defined stress-fibers. Whilst both MC3T3-E1(14) (64) and MG63 (65) monolayer cultures show fibroblastic morphology during logarithmic growth *in vitro*, they assume a pyriform shape with prominent stress-fibers across their cell bodies when confluent (39, 64). *In vivo*, osteoblasts can be ovoid, rectangular, columnar, cuboidal, or pyriform (66). Osteoblasts form a pavement-like or “overlapping roof tiles” monolayer on the bone surface [Bidder, 1906 as cited in Bourne (66) and Sudo et al. (64)] overlaying osteocytes within the bone matrix [Gegenbaur, 1864 as cited in Bourne (66)]. Osteoblast position is essential for osteocyte–osteoblast interactions, which ultimately regulate bone matrix formation (36, 67–69). Osteoblast morphology in the 3D co-culture is thus consistent with *in vitro* and *in vivo* observations.

In the 3D co-culture model, MLO-Y4 cells maintain their osteocytic morphology throughout all gel depths for 7 days, with cell projections from adjacent cells in contact. *In vivo*, osteocytes present a dendritic morphology that allows communication with neighboring osteocytes. This forms an extensive network known as the LCS (12, 70–73), which permits metabolic traffic and exchange within the mineralized environment of the bone matrix. *In vitro*, monolayer cultures of MLO-Y4 cells display a 2D dendritic morphology, which becomes 3D in collagen gel cultures (34, 39). Furthermore, IDG-SW3 cells also display dendritic morphology in 3D gels (35). The osteocyte morphology in the 3D co-cultures is consistent with both *in vivo* and *in vitro* observations, with morphological characteristics indicative of a 3D network throughout the co-culture.

### Osteocyte and osteoblast phenotype in 3D co-cultures

In 3D co-cultures, MC3T3-E1(14) cells expressed E11 mRNA and protein and MG63 cells expressed E11 mRNA (antibody does not recognize human E11). E11 has been detected in mature osteoblasts and osteoblasts undergoing bone matrix synthesis (74–78). The E11 expression detected in the surface zone of the model suggests that osteoblasts may be sending out projections to connect with neighboring osteoblasts and/or embedded osteocytes. Previous studies have shown that osteoblasts have cytoplasmic processes connecting them to neighboring cells [Spuler, 1899 as cited in Bourne (66), Wetterwald et al. (75), and Schulze et al. (79)], with prominent stress-fibers that stretch across their cell bodies into small cytoplasmic processes (39).

OCN, Runx2, and *Col1a1* were also expressed in MC3T3-E1(14) and MG63 cells when in 3D co-cultures, consistent with *in vivo* and *in vitro* studies (51, 80–84). ALP mRNA expression was also found in MC3T3-E1(14) cells in 3D co-cultures consistent with previous reports (51, 80, 81, 84–86). CX43 protein expression was observed throughout osteoblast cytoplasm and cell membrane in both MC3T3-E1(14) and MG63 cells in 3D co-cultures, suggesting that the surface osteoblasts have the potential to connect to neighboring cells. Osteoblasts have been shown to express the gap junction CX43 protein (36, 87, 88), which is important in responses to mechanical loading (21) and skeletal function (68, 89, 90).

In 3D co-cultures MLO-Y4 cells expressed mRNA and protein for the early osteocyte marker E11 (75–77, 79) consistent with previous data (75–77). OCN, Runx2, *Col1a1*, and ALP mRNAs were also detected in these cells in co-cultures. Osteocytes have been previously shown to express OCN, *Col1a1*, and ALP *in vivo* and *in vitro* (34, 91) and Runx2 *in vitro* (92).

Confocal imaging of osteocytes in 3D co-cultures showed the presence of CX43 throughout the cytoplasm, osteocytic processes, and around the nucleus. Osteocytes express CX43 both *in vivo* and *in vitro* (34, 36, 93, 94), which allows the formation of the LCS within the bone matrix, and connects osteocytes to surface osteoblasts (36, 95). CX43 gap junctions in osteocytes contribute to bone remodeling and formation (96) and they are also mediate load-induced PGE_2_ release in osteocytes (97). The expression of CX43 indicates that osteocytes within the 3D co-culture are potentially able to form a network similar to the LCS, as well as connect to the osteoblasts on the surface.

In 3D co-cultures, MC3T3-E1(14) cells showed a significantly higher expression of *Col1a1* mRNA compared to the deep zone of the model. Interestingly, deep zone cells within the 3D co-cultures expressed significantly higher levels of ALP compared to the surface zone of the model. This suggests that under appropriate conditions the MLO-Y4 cells within the 3D model may contribute to mineralization the collagen matrix within which they are embedded, as previously seen in embedded IDG-SW3 cells (35).

### Testing osteogenic responses in the 3D co-culture model

Pilot experiments were performed to see whether the co-culture methodology could reveal an osteogenic response. This was tested firstly by treatment with BMP-2 and secondly by testing responses to mechanical loading.

In the MLO-Y4/MG63 co-culture, osteoblasts were able to respond to BMP-2, by significantly increasing their *COL1A1* mRNA expression and showing abundant type I pro-collagen protein expression after 5 days of BMP-2 treatment. These data are consistent with previous *in vitro* studies, which showed that BMP-2 stimulates collagen synthesis in MC3T3-E1 cells (85).

To determine whether the osteocytes within the co-culture model responded to loading, we cultured MLO-Y4 in 3D collagen gels, without surface osteoblasts, and measured PGE_2_ release in response to loading. To facilitate loading of the 3D model, a 16-well silicone plate was developed that applied uniform strain within each gel. The loading regime applied (5 min, 10 Hz, 2.5 N) was based on previous publications showing that 10 min of 10 Hz, 4000–4500 με loading is physiological and osteogenic *in vivo* (91, 98, 99). In 3D osteocyte mono-cultures, loading induced PGE_2_ release over 24 h with maximum PGE_2_ release occurred after 0.5 h. In osteocytes pre-cultured in 3D collagen gels for 48, 72 h, or 7 days, mechanical loading increased PGE_2_ release 0.5 h post-load. No PGE_2_ release occurred in osteocytes pre-cultured in 3D gels for 24 h. This suggests that the osteocytes may require at least 48 h in 3D collagen gels to develop an osteocytic phenotype, form dendrites and the CX43 gap junctions that are involved in the release of PGE_2_ from osteocytes *in vitro* (100, 101). Others have shown that mechanically loaded osteocytes in monolayer increase PGE_2_ release (24, 93, 102, 103), as early as 0.5 h post-load (93) but no previous studies have investigated osteocyte response to load in 3D.

To determine whether mechanical loading in 3D co-cultures could elicit an osteogenic response, co-cultures were mechanically loaded as before and type I collagen synthesis quantified. In 3D co-cultures, mechanical loading increased PINP release, suggesting that mechanical stimuli of 3D co-cultures elicit an osteogenic response. PINP synthesis was measured from whole 3D co-cultures, therefore, PINP synthesis may not only be from surface osteoblasts, but also from embedded osteocytes. Both osteoblasts and osteocytes produce type I collagen *in vitro* (34, 104) although MLO-Y4 cells express reduced *Col1a1* mRNA compared to osteoblasts both in monolayer (34) and here in 3D co-cultures.

Our preliminary data showing that both BMP-2 and mechanical loading can induce type I collagen synthesis, reveals the potential for the new 3D co-culture and loading methodology described in this paper in investigating osteogenic responses regulated by osteocytes.

### Limitations of the 3D co-culture model

#### Cell migration in co-cultures

The 3D co-culture method is subject to the possibility of cross-contamination of RNA between surface osteoblasts and embedded osteocytes, due to the extraction protocol, or mixing of cell types between zones due to osteoblast and/or osteocyte migration. We used expression of the SV40 large T-antigen, exclusive to MLO-Y4 cells [derived from mice expressing the SV40 large T-antigen oncogene under the control of the OCN promoter (34)], and an antibody that detects human but not mouse type I pro-collagen, to investigate this. The expression of SV40 large T-antigen mRNA in RNA extracted from the surface zone, suggests that there is low level RNA cross-contamination from the osteocytes, or MLO-Y4 cell migration to the surface in MLO-Y4/MC3T3-E1(14) co-cultures. Since no SV40 large T-antigen immunostaining was observed in the surface zone of the model even after 7 days of co-culture, we conclude that no osteocytes migrated to the surface zone of the 3D co-culture and that the SV40 large T-antigen mRNA contamination in the surface zone is due to MLO-Y4 cells immediately underlying gel surface, lysing, and releasing RNA during extraction from surface cells. However, the human-specific type I pro-collagen antibody revealed that, although rare, some MG63 cells migrated from the surface to the deep zone of the model, in MLO-Y4/MG63 co-cultures.

#### Phenotype and function

Whilst the 3D co-culture model described here has shown osteoblast and osteocyte cell viabilities (58–60, 105, 106), morphologies (66, 71, 107, 108), phenotypes (75, 81, 86, 91, 109–114), and loading (104) and osteogenic responses (115, 116) consistent with those found *in vivo*, the use of MLO-Y4 cells means that the important mechanically regulated factor, SOST, is not expressed in the osteocytes in the model. This limitation could be solved by replacing the MLO-Y4 cells with the IDG-SW3 cell line, which are able to differentiate into mature osteocytes and express SOST (35). Furthermore, the phenotypic characterization was performed in 3D co-cultures grown in plastic plates and it is possible that aspects of the phenotype would be affected by growing cells in silicone plates.

Although the 3D model is designed to investigate mechanically induced osteogenesis in a similar *in vivo* physiological environment, it is not mineralized and so it would only represent interactions that occur in newly formed osteoid rather than mineralized bone. Previous studies have shown the mineralization of 3D collagen gels is possible with IDG-SW3 cells (35) during differentiation to osteocytes, and therefore the 3D co-culture could be mineralized. Mineralization of the 3D collagen gel would affect the properties of the matrix and cell–ECM interactions. Previous *in vitro* studies have shown that after mineralization the ECM of monolayer cultures became gradually stiffer (117). Furthermore, ECM composition has been shown to affect gene expression (118) and osteoblast differentiation and behavior (119). Therefore, mineralizing the 3D co-culture would make the collagen gels stiffer and alter phenotype, further mimicking a physiological environment. If the 3D co-culture was mineralized, further investigations should be done to test the mechanical properties of the mineralized 3D co-cultures as well as the viability and phenotype of the cells within the model and assess whether the medium nutrients can still diffuse to all areas of the 3D gel.

A technical challenge is to ensure that MLO-Y4 cells are evenly distributed within the collagen solution when gels are being set up, otherwise some 3D cultures will have more osteocytes than others. This will lead to cell number variability between experimental replicates, which could cause differences in osteocytic network and loading responses. This was further affected by a variation of up to 20% in the weight of collagen supplied by Sigma, meaning that, since the defined collagen mass was dissolved in a set volume of acid, the collagen gels varied from 2.0 to 2.6 mg/ml. These are potential explanations for differences in magnitude of responses across independent experiments and the essential 3D pre-culture time of at least 48 h for a consistent increase in PGE_2_ in response to mechanical loading.

#### Mechanical loading device

Currently, there are two devices similar to the one developed here (120, 121). Tata et al. (121) developed a silicone plate in a six-well plate format to mechanically load vascular smooth muscle cells (VSMCs) in monolayers, whereas, the device developed by Neidlinger-Wilke et al. (120) is a single-well silicone plate, which was designed to load 3D collagen cultures of intervertebral disk cells. Both devices applied cyclic mechanical stimuli by stretching in a similar fashion to the device described here, but used much higher strains at low frequency (Neidlinger-Wilke et al., 24 h, 0.1 Hz, 10,000 με; Tata et al., 6–72 h, 1 Hz, 10–20% strain) (120, 121). Neidlinger-Wilke et al. (120) did not publish how they assessed strain associated with their device. Tata et al. (121) assessed the strain field at the bottom surface of the wells using finite element (FE) modeling, but did not validate this FE model with DIC, or any other methods. Therefore, our loading device is the first where the strains have been directly measured, albeit on the plate surface rather than within the gel.

Digital image correlation showed that when 2.5 N is applied to the silicone plate, the majority of the wells experienced strains of 4000–4500 με. Peak strain values in vertebrate bone range from 2000 to 3500 με (122–125), 4000–4500 με loading is physiological and osteogenic (91, 98, 99), whereas, 6000 με is pathophysiological (126). The strain testing performed was carried out on an empty plate. Testing a silicone plate with 3D cultures within the wells would further validate the loading plate. Whilst incorporation of particles into the 3D gels (127) would enable strains to be measured directly within the gels, we were unable to achieve this by DIC given the limited well size and the pink color and reflective properties of the gels. Further work is necessary to confirm the strain experienced by the cells in the gels is similar to that on the base of the plate.

## Conclusion

There is a great need for a fully characterized *in vitro* 3D matrix based bone model. The majority of the available 3D models involve culturing cells on scaffolds (44–46, 128), which does not represent the bone environment *in vivo* where osteocytes, are embedded within a matrix. Published models involving embedding osteoblasts (39, 129), MLO-Y4 (38, 39), primary osteocytes (42), or normal human bone-derived cells (NHBCs) (41) within a matrix showed maintenance of cell viability (38, 129), osteocyte cell morphology (38, 39, 41, 42), connectivity (38), and gene expression (41, 42). However, none of these models have been individually assessed in all key areas. Furthermore, none of the available 3D collagen based cultures involve co-culturing osteocytes and osteoblasts, nor they have been exposed to mechanical stimuli. Therefore, none investigate the important interactions between these cell types, which lead to mechanically induced bone formation.

This co-culture model facilitates a 3D network of osteocyte-like cells that can be subjected to appropriate anabolic and mechanical loading cues to act upon osteoblasts. Osteoblasts and osteocytes retain appropriate morphology, phenotype, and viability, and osteoblasts increase *COL1A1* expression when stimulated with BMP-2 and mechanical load. Furthermore, embedded osteocytes respond to mechanical loading by releasing PGE_2_. Potentially, this model may be useful in elucidating osteocyte-driven mechanisms that regulate bone formation as a result of mechanical loading, something other current 3D models do not provide (38, 39, 41, 42). The 3D co-culture, combined with a multi-well loading system could provide a novel platform for drug discovery and development for the treatment of age-related bone diseases.

## Author Contributions

Conception and design: Marisol Vazquez, Bronwen A. J. Evans, Daniela Riccardi, Sam L. Evans, Jim R. Ralphs, Christopher M. Dillingham, Deborah J. Mason. Collection and assembly of data: Marisol Vazquez, Sam L. Evans, Jim R. Ralphs, Christopher M. Dillingham. Analysis and interpretation of data: Marisol Vazquez, Bronwen A. J. Evans, Daniela Riccardi, Sam L. Evans, Deborah J. Mason. Drafting of the manuscript: Marisol Vazquez, Deborah J. Mason. Critical revision: Marisol Vazquez, Bronwen A. J. Evans, Daniela Riccardi, Sam L. Evans, Deborah J. Mason. Final approval of the article: Marisol Vazquez, Bronwen A. J. Evans, Daniela Riccardi, Sam L. Evans, Jim R. Ralphs, Christopher M. Dillingham, Deborah J. Mason.

## Conflict of Interest Statement

The authors declare that the research was conducted in the absence of any commercial or financial relationships that could be construed as a potential conflict of interest.

## Footnotes

^1^http://www.mdl.dk/publicationsnormfinder.htm

^2^http://www.technovent.com

^3^http://www.snazaroo.co.uk

^4^http://www.limess.com
